# Type I interferon signaling in SARS-CoV-2 associated neurocognitive disorder (SAND): Mapping host-virus interactions to an etiopathogenesis

**DOI:** 10.3389/fneur.2022.1063298

**Published:** 2022-12-07

**Authors:** George D. Vavougios, Gabriel A. de Erausquin, Heather M. Snyder

**Affiliations:** ^1^Department of Neurology, University of Cyprus, Lefkosia, Cyprus; ^2^Department of Respiratory Medicine, University of Thessaly, Larisa, Greece; ^3^The Glenn Biggs Institute for Alzheimer's and Neurodegenerative Diseases, UTHSA, San Antonio, TX, United States; ^4^Division of Medical and Scientific Relations, Alzheimer's Association, Chicago, IL, United States

**Keywords:** SARS-CoV-2, cognitive impairment, tauopathy, type I interferon signaling, host-virus interaction, dementia

## Abstract

Epidemiological, clinical, and radiological studies have provided insights into the phenomenology and biological basis of cognitive impairment in COVID-19 survivors. Furthermore, its association with biomarkers associated with neuroinflammation and neurodegeneration supports the notion that it is a distinct aspect of LongCOVID syndrome with specific underlying biology. Accounting for the latter, translational studies on SARS-CoV-2's interactions with its hosts have provided evidence on type I interferon dysregulation, which is seen in neuroinflammatory and neurodegenerative diseases. To date, studies attempting to describe this overlap have only described common mechanisms. In this manuscript, we attempt to propose a mechanistic model based on the host-virus interaction hypothesis. We discuss the molecular basis for a SARS-CoV-2-associated neurocognitive disorder (SAND) focusing on specific genes and pathways with potential mechanistic implications, several of which have been predicted by Vavougios and their research group. Furthermore, our hypothesis links translational evidence on interferon-responsive gene perturbations introduced by SARS-CoV-2 and known dysregulated pathways in dementia. Discussion emphasizes the crosstalk between central and peripheral immunity *via* danger-associated molecular patterns in inducing SAND's emergence in the absence of neuroinfection. Finally, we outline approaches to identifying targets that are both testable and druggable, and could serve in the design of future clinical and translational studies.

## Introduction

Cognitive impairment secondary to COVID-19 is now a recognized, health concern. It emerges as part of the LongCOVID spectrum, without a clearly defined cause (1). Clinical, pathological and radiological manifestations of this SARS-CoV-2 associated neurocognitive disorder (SAND) have outlined its significant overlap with neurodegenerative dementia (2), which extends to biomarkers in some individuals to include biomarkers consistent with neurodegenerative diseases such as Alzheimer's disease (AD), including beta amyloid oligomers (Aβ), tau, neurofilament light chain (Nfl) and others (3, 4). Towards this end, several recent translational studies have confirmed overlap on the molecular level of contributing biology between COVID-19 and AD disease, with innate immunity at its epicenter (5–12). Collectively, these studies point toward type I interferon signaling, a pathway contested by SARS-CoV-2 (13), as the potential culprit. Furthermore, interferon responsive genes such as those in the ISG, OAS, and IFITM families, dysregulated by SARS-CoV-2, have recently and independently emerged as key players in AD (9, 14, 15). To date, studies attempting to summarize the evidence on this overlap have not attempted to explore their synthesis towards an etiopathogenic mechanism emerging from host-virus interactions.

This review aims to outline emerging evidence on the genes and pathways that could define SAND on the molecular level. We aim to go beyond a presentation of potential mechanisms, presenting them instead through the evolution of host-virus interactions, the mobilization of innate immunity, and the consequences of both.

## The viral lifecycle: Kinase recruitment and tauopathy

The first specific mechanistic indication that the intracellular lifecycle of SARS-CoV-2 may be linked with neurodegeneration, and specifically with tauopathies, came from a brain organoid infection model; SARS-CoV-2 neuroinfection was quiescent, causing neuronal apoptosis with hyperphosphorylated tau as its hallmark (16). A possible explanation for these findings is that SARS-CoV-2-dependent perturbations in kinases such as FYN (10) and GSK3 (17, 18) during their recruitment as part of the virus' lifecycle could escalate to increasing downstream tau hyperphosphorylation and oligomerization, as seen in other RNA viruses, i.e. DENV (19) and HIV-1 (20). In the setting of the human central nervous system (CNS), the mechanism of tau hyperphosphorylation and oligomerization, however, may not require subsequent *de novo* infection. Rather, increasing evidence suggests that transsynaptic spread of tau (21, 22), amyloid oligomers (8), and viral particles *via* extracellular vesicles (5) may sustain a neuroinflammatory process from an infected hub and this may evolve to or enhance pre-existing neurodegeneration in its connected network (23, 24).

The combination of anosmia, cognitive impairment, and limbic degeneration in some individuals suggests a link between SAND and neurodegenerative dementia (25) and with tau pathology specifically (26). In humans, significant differences in peripheral markers of age-related neurodegeneration, including specific forms of phosphorylated tau or p-tau have been identified both in COVID-19 patients (27) and survivors in the post-COVID-19 setting over 6 months follow up (28). Notably, these changes appear linked with proinflammatory cytokines, however not all data show that these are persistent (3) and there is still much to learn about the biological underpinnings that may continue to contribute.

Taken together, both phenomenology, biomarkers, and underlying genes potentially recruited by SARS-CoV-2 indicate that tauopathy may be a plausible mechanism by which the CNS is affected. Notably, the transmission of tau seeds *via* peripheral sites to the CNS *via* exosomes and their neurotoxicity has been previously observed in *P. Aeruginosa* pneumonia (29), furthermore indicating that systemic infection may affect the CNS even in the absence of neuroinfection. Considering that tau can activate type I interferon signaling as seen in neurodegenerative disease in the absence of infection (30), tau transmission during SARS-CoV-2 infection could be seen as a canonical alarmin/pathogen-associated molecular pattern (PAMP) (31–33), which can readily lead to a detriment for the recipient cell.

### The host response: Type I interferon response, amyloid beta, and cognitive impairment

Type I interferon (IFN-1) perturbations are an established hallmark of Alzheimer's disease, mediating neuroinflammatory synapse loss (14, 34). During SARS-CoV-2 infection, IFN-I pathways are among the first activated pathways between host and pathogen, a finding confirmed by multiple translational studies (13, 35). From then on, the interaction between IFN-I signaling, a canonical response to infection (36), and SARS-CoV-2's immunoevasion stratagems are highly complex (37). As a primary event, SARS-CoV-2's lifecycle may be effectively disrupted by a pre-established IFN-I cellular milieu (38). On the contrary, delayed type I responses in the nasal epithelial have been shown to enhance SARS-CoV-2 permissiveness (39). Correspondingly, inborn errors in IFN-I may render carriers specifically vulnerable to SARS-CoV-2, as they correspond to differentially perturbed IFN-I responses (40, 41). Adding to the complexity of this interaction is SARS-CoV-2's armamentarium of proteins that target IFN-I responses (42). Notably, these same targets of virus-host protein interactions also play a central role in neuroinflammation and neurodegeneration, for example in TBK1 (42, 43), KPNA2/Karyopherin (44, 45), and alpha-synuclein, among others.

Further dissection of the IFN-I signalosome reveals specific genes that are key players in both innate immune responses and AD. In recently published work by Vavougios and colleagues (46), disrupted proteostasis and trained immunity pathways were among overlapping molecular pathways that are common across different tissues in AD. This work suggested that IFN-1 has a specific relationship with unique signaling cascades, focusing on the IFN response to antiviral effectors, such as the interferon-inducible transmembrane (IFITM) and the 2′-5′-oligoadenylate synthase (OAS) family genes in both AD and COVID-19 (15). The specific relationship of perturbed IFN-I signaling to both AD and COVID-19, focuses on interferon responsive antiviral effectors, such as IFITMs and OASs, which are interferon stimulated (ISGs) gene families that provide cellular-level defense against intracellular pathogens. Dysfunctions of IFITMs and OASs on a pathway level not only have the potential to abrogate antiviral activity, but several studies suggest this dysfunction enables these factors to act as pro-viral factors (47–49). Vavougios and colleagues found that IFN-I signatures containing members of these ISG families are common in neurons, peripheral immune cells, and microglia affected by COVID-19 or by AD (10, 15, 33, 50, 51).

The relationship of both gene families, as well as other ISGs such as MX and IFITs (10, 15), and IFN-I signaling as a nexus for both COVID-19 and AD has been corroborated by others in various experimental and model system settings (6, 7, 9, 11–13, 52–54). Gamma secretase activity in response to viral infection has also been shown to be functionally linked with type I and type II interferon responses in peripheral immune cells; gamma secretase is involved in the production of the beta-amyloid protein (55). Lastly, IFN1 signaling in AD-related microglia was shown to upregulate IFITM3, which in turn modulates gamma secretase processing. The antigenic stimulus for this cascade of molecular events was nucleic acid (NA)-enriched neuritic plaques, and notably, microglia may not then distinguish viral from endogenous NAs (14, 49). This suggests that as an innate immunity protein, IFITM3 may canonically intercept SARS-CoV-2 (56), with its upregulation concomitantly building up to both increased beta amyloid production (14) and fed-forward IFN-I upregulation (34). Notably, such interactions have also been observed with the structurally similar IFITM2 in modulating the host's type I interferon signaling.

Taken together, these events show that IFN-I signaling dysregulation secondary to SARS-CoV-2 infection may be relayed by endothelial cells (7, 11, 54) to microglia, priming them (57) and may potentially result in upregulation of IFITMs and increased presence of beta amyloid production (57). If this priming is successful in restricting SARS-CoV-2, as heralded by S1 – Aβ_1 − 42_ interactions (58), neuroinflammation but not neuroinvasion would be expected to predominate. Notably, S1 itself has been shown to function as a danger-associated molecular pattern (DAMP) for microglia, furthermore inducing neuroinflammatory phenotypes (32, 57), indicating that Roy et al.'s (49). HSV-1 model of AD pathogenesis may also provide some context to consider for SARS-CoV-2 (59). Furthermore, in the same model, the transmission of tau seeds as observed elsewhere (29) would also fit our current understanding of tau and Aβ as Type I interferon stimulants, as observed in neurodegenerative disease (30).

### SARS-CoV-2 associated neurocognitive disorder as innate immunity's pyrrhic victory

Regardless of the specific pathogen or PAMP (33) involved, IFN1 signaling canonically induced as an innate immunity response is a firmly recognized inducer of cognitive impairment (34, 55, 60). Specific molecular events that may account for this relationship involve increased beta amyloid production, proinflammatory microglial activation, and impaired neuronal homeostasis (14, 34, 49, 60). SARS-CoV-2 introduction to this system is an immunogenic challenge with potential advanced capabilities to modulate IFN-I signaling, subverting it to its favor processes that enable evasion of the immune system (13). An example of this proposed mechanism can be found in the amyloidogenic interaction between N and alpha-synuclein (aSyn), where N functions as a scaffold for aSyn aggregation (61). The abrogation of aSyn would arrest its function as a canonical, neuron-specific IFN-I modulator (62); the aggregation of aSyn however would in turn activate IFN-I by a (presumably) non-canonical pathway, observed in neurodegenerative disease (63). This sterile proinflammatory signal could be relayed centrally from infected microvascular endothelia or olfactory epithelial cells, to be intercepted primarily by microglia (7, 11, 57). Aside from aSyn specifically, interactions between SARS-CoV-2 proteins and other proteopathic seeds. Notably, as per a previous model proposed by Vavougios et al. (15), the neuroanatomical premise of this concept is supported by imaging data indicating tandem degeneration of entorhinal cortex and hippocampi (25) and murine models of intranasal administration of SARS-CoV-2 that develop late onset proteinopathy, even after viral clearance (57, 59). Furthermore, our model's main premise, i.e. the capability of SARS-CoV-2 protein fragments to induce amyloidogenesis and subsequent neuroinflammation is confirmed in at least one *in vitro* model (64).

Lytic replication or multiple infected sites may not be required for cognitive impairment to manifest, along with molecular events similar to those of neurodegenerative dementias. Successful restriction *via* IFN-I and feed-forward signaling is still impacting the CNS, fully capable of establishing neuroinflammation, proteinopathy, and microgliosis in the absence of a pathogen (57, 59, 64) building up to synapse loss (34, 49). From an immune perspective, however, this destruction proximal to an infected site successfully walls off an invading pathogen, being informed by both IFN-I and exosomal tau, here functioning as evidence of viral latency (16, 20, 21). Of note, once initiated, the overproduction of beta amyloid was shown to enhance the capability of native molecules to activate microglia and initiate IFN-I cascades (49). This notion indicates that both different pathogens targeting IFN-I (55), Danger-associated molecular signals (DAMPs) (32) and self-DAMPs (34, 49), accumulated by failing organelles and defects in proteostasis and mitochondrial homeostasis, may readily activate this pathogenetic mechanism in the absence of an exogenous immune challenge. Considering that IFN-I may be targeted by the viral lifecycle and successfully suppressed, second-order or non-canonical as described herein activation of IFN-I by the very same “captured” molecules (i.e. aSyn, tau, Aβ) would serve as a failsafe. Notably, the sterile enhancement of microglial IFN-I cascades has been previously shown (34, 49, 62, 63) indicating that their enhancement in the setting of SARS-CoV-2 (61, 65) infections may require proteins or DAMPs rather than a complete virion—a concept that would account for the persistence of neuroinflammation past virus clearance (59).

## Conclusions

The SARS-CoV-2 pandemic has provided a forum to better understand the contributions of recurrent and agnostic immunity in response to some pathogen exposure rather than specific exposure and its relationship to AD-specific biology (22). AD is a complex disease, and likely has a number of factors that contribute to later life risk. There are many outstanding questions and in future studies, SAND-related contributions should be considered within the potential limitations.

As a standalone syndrome, the SARS-CoV-2 associated neurocognitive disorder (SAND) poses an interesting question: is the salience of COVID-19, increased population exposure, and potent induction of IFN-I the true culprit? Prior to SARS-CoV-2, HIV-1 and its Tat protein had been shown to intersect with both tau and beta-amyloid and potentially engage with the AD molecular pathology (20), and a corresponding HIV-1 associated neurocognitive dysfunction (HAND) associated with infection. SAND, much like HAND before it, indicates the long-standing impact of a pathogen may be as impactful for the individual as the native infection, when inflammation is either unmitigated, self-propagating, or both.

While these emerging links between neuroinflammation, neurodegeneration, and COVID-19 represent a growing body of literature, it is important to underscore that the natural history of cognitive, functional, and behavioral defects in individuals experiencing long-term neurological sequelae is unknown. There are many unanswered questions about the linkage, and it is important to understand whether translational models and clinical radiological entities represent a clear, mechanistic continuum. Furthermore, it is not yet known if COVID-19's effects on cognition represent lasting or transient impairments. It is also not known why some individuals experience long-term impact on their cognition, function, and behavior, while others do not. COVID-19's effect on cognition should also be considered multifactorial, considering its implication in vascular damage to the brain and sleep-related complaints affecting survivors (66). Furthermore, the introduction of vaccines may provide information on how these biological underpinnings interact with one another.

In this review, we offer a potential model for SAND following the trail of host-virus interactions and combining it with the dual roles of proteopathic seeds as DAMPs/PAMPs and IFN-I signaling and propose a framework to further extend these findings to linkages with neurodegenerative disease. Building upon previous works from Vavougios et al. and others, this manuscript outlines a potential opportunity to formulate a working, testable hypothesis on SAND with implications on cognitive impairment and other dementias. Furthermore, as we have previously indicated, we outline targets that are both testable and druggable (51), and could serve in the design of future clinical and translational studies.

The global research and clinical communities must continue to work together to uncover the answers to these, as well as other, questions on the intersection of COVID-19, the brain, and neurodegeneration.

## Author's note

The authors are all participants in the Alzheimer's Association SARS-CoV-2 Consortium on Neurological Sequelae and continue to collaborate to better understand the longterm neurological implications of COVID-19.

## Author contributions

All authors listed have made a substantial, direct, and intellectual contribution to the work and approved it for publication.
